# Transcript Profiling and Gene Identification Involved in the Ethylene Signal Transduction Pathways of Creeping Bentgrass (*Agrostis stolonifera*) during ISR Response Induced by Butanediol

**DOI:** 10.3390/molecules23030706

**Published:** 2018-03-20

**Authors:** Han-Yu Jiang, Jin-Lin Zhang, Jiang-Wei Yang, Hui-Ling Ma

**Affiliations:** 1 Pratacultural College, Gansu Agricultural University, Lanzhou 730070, China; jianghy@gsau.edu.cn; 2 Key Laboratory of Grassland Ecosystem, Ministry of Education, Lanzhou 730070, China; 3 Sino-U.S. Center for Grazingland Ecosystem Sustainability, Lanzhou 730070, China; 4 College of Life Science and Technology, Gansu Agricultural University, Lanzhou 730070, China; jlzhang@lzu.edu.cn (J.-L.Z.); Yangjw@gsau.edu.cn (J.-W.Y.); 5 College of Pastoral Agricultural Science and Technology, Lanzhou University, Lanzhou 730000, China

**Keywords:** creeping bentgrass, transcriptome, ethylene, ACO, ACS, ISR response, *Rhizoctonia solani*, butanediol

## Abstract

Creeping bentgrass (*Agrostis stolonifera*) is the preferred green lawn grass, with excellent turf characteristics but poor disease resistance. At present, the mechanisms of disease resistance in creeping bentgrass are poorly understood, especially the ethylene signal transduction pathway under the induced systemic resistance (ISR) response. In this study, butanediol (BDO), as a new type of disease-resistance compound, was applied to creeping bentgrass seedlings to induce the ISR response. Then, we measured ethylene production and related enzyme activities. Additionally, transcript profiling and gene identification were performed in association to ethylene signal transduction pathways. The changes of ethylene production and related enzyme 1-aminocyclopropane-1-carboxylic acid oxidase (ACO) and 1-aminocyclopropane-1-carboxylic acid synthases (ACS) activities showed significant difference at 24 h after *Rhizoctonia solani* inoculation among five treatments of various BDO concentrations. After 100 µmol L^−1^ BDO treatment, ethylene production and related enzyme activities reached their peak levels. Additionally, 208,672 unigenes of creeping bentgrass were obtained by de novo assembly. In total, 15,903 annotated unigenes were grouped into 33 canonical pathways in the KEGG (Kyoto Encyclopedia of Genes and Genomes) analysis. Among those, 1803 unigenes were classified as ‘signal transduction’. There were 6766 differentially expressed genes (DEGs) among B24 (inoculated-rhizobacteria in MS medium with 100 µmol L^−1^ BDO for 24 h), NB24, B72 and NB24 (no rhizobacteria in MS medium with 100 µmol L^−1^ BDO for 24 h) libraries, and 4,639 DEGs between B24 and B72 (inoculated-rhizobacteria in MS medium with 100 µmol L^−1^ BDO for 72 h) libraries, with 4489 DEGs in all three libraries. As suggested by the RT-PCR assay, the expression levels of ethylene-responsive and defense-related genes were variable among treated samples during the BDO-induced ISR responses. The expression levels of *EIN, ERF*, *NPR1*, *PR3* and *PR4* genes increased and reached their peaks in the first 24 h after *R. solani* infection in the BDO-induced ISR reaction compared with NB24 treatments. This results is consistent with the changes of important ethylene biosynthetic enzymes and ethylene concentrations during the BDO-induced ISR responses. We further found the intermediate substances for the signaling pathway, and the relationships between the expression levels of BDO-induced ISR disease-resistance genes and those of the response genes for ethylene signal pathway. Our findings present a genetic basis for systemic resistance of creeping bentgrass through transcriptomic analysis and our study provides a theoretical and practical basis for the improvement of turfgrass disease resistance and quality.

## 1. Introduction

Creeping bentgrass (*Agrostis soionifera*) is a perennial herb of the family Gramineae and belongs to the cool-season turfgrasses. Because of its strong adaptability, drought resistance, soft and delicate blades, rapid growth and strong ornamental characteristics, it was widely used on golf course fairways, grass tennis courts, courtyards and parks [1,2,3]. However, creeping bentgrass has poor disease resistance and is easily infected by various diseases, including dollar spot, downy mildew, snow rot and smut, which causes serious decreases in turf quality and function, resulting in huge losses to turf production and management. For a long time, the resistant varieties and chemical fungicides have been used as prevention methods against lawn diseases [4]. However, the traditional breeding cycle is too long. Most of the control effects of chemical fungicides are not ideal, and chemical pesticide accumulation in the soil will seriously pollute the environments. Thus, a new effective and environmentally friendly way to control the diseases is to use an inducer to induce disease resistance in turfgrass. Butanediol (BDO) is a new type of disease resistance-inducing factor, which has no toxicity, produces no pollution and provides durable disease resistance. Cortes-Barco and Hsiang reported the induction of disease-resistance in creeping bentgrass for the first time, and ISR produced by BDO effectively inhibited grass leaf diseases [5]. Subsequently, BDO can induce disease-resistance in creeping bentgrass, and resistance effect is greater than that of the systemic acquired resistance (SAR) pathway by benzothiadiazole (BTH) induction [6].

Plants induce an innate immunity that is highly flexible and involves a complex response mechanism that recognizes and counteracts different invaders. At first, plants can use their own physical and chemical barriers, and then activate some inducible defensive mechanisms. At present, several systemic defensive response mechanisms are known, such as SAR, which is activated by pathogens producing limited infections, for example, hypersensitive necrosis response [7]; another is ISR, which is elicited upon the infection of roots by selecting a series of non-pathogenic rhizobacteria [8,9]. The ISR responses were found in several plants, such as *Arabidopsis thaliana*, carnation, legumes, cucumber and potato [10]. It has a broad-spectrum resistance to fungi, bacteria and viruses. The resistance to *Rhizoctonia solani* in rice was activated by FP7 and PF1 *Pseudomonas fluorescens* strain [11]. Additionally, in creeping bentgrass, the *P. fluorescens* HP72 strain induced resistance to *R. solani* [12].

Ethylene plays roles in regulating several aspects of plant development, including fruit ripening, seed germination and leaf senescence. In addition, ethylene also has relation to responses to abiotic and biotic stresses. Ethylene plays a key role and responds to diverse types of induced resistances against pathogens through complex networks. Furthermore, recently, the important components in the ethylene-signaling pathway related to disease resistance were revealed by genetic analysis. As a signal molecule to transcription factors, ethylene activates downstream gene expression [13,14,15,16]. Thus, ethylene is also an important signal molecule in ISR. The ISR induced by Rhizobacteria is practically always dependent on jasmonic acid (JA) and ethylene (ET) signal [9]. In the ISR system, ethylene-regulated processes are initiated by ethylene synthesis, in which S-adenosylmethionine is changed into 1-aminocyclopropane-1-carboxylic acid (ACC) by the enzyme ACC synthase (ACS). Then ACC is changed to ethylene by ACC oxidase (ACO). Following these processes, ethylene and its membrane-bound receptor proteins regulate the downstream central regulatory protein EIN2, which trigger the expression of diverse transcription factors. These factors activate downstream gene expressions, including those of some protection genes and ethylene responsive genes [17,18]. In *Arabidopisis* plants expressing WCS417r-mediated ISR, a series of ET-and/or JA-responsive defense-related genes, such as *VSP2*, *PDF1.2*, *LOX1*, *LOX2, PAL1*, *CHIB*, and *HEL*, were also expressed [19]. Presently, our comprehension about the signaling constituents in ethylene-biosynthetic downstream, the ethylene-signal pathways which change biosynthesis under disease stress, and especially ISR response mechanisms, are largely unknown. Thus, in our study BDO was applied to roots of creeping bentgrass as a new type of disease-resistance compound to induce ISR disease-resistant responses. Our objectives were: (i) to lay a foundation in genetic level for the creeping bentgrass transcriptomic analysis; (ii) to analyze ethylene-dependent signal transduction pathways involved in ISR mechanisms; and (iii) to find the intermediate substances in the signaling pathway and the relationships between the BDO-induced ISR disease-resistance genes and the response genes of the ethylene signal pathway.

## 2. Results

### 2.1. Ethylene Production and Related Enzyme Activities in Creeping Bentgrass under the ISR Response Were Induced by BDO

**Figure 1:**
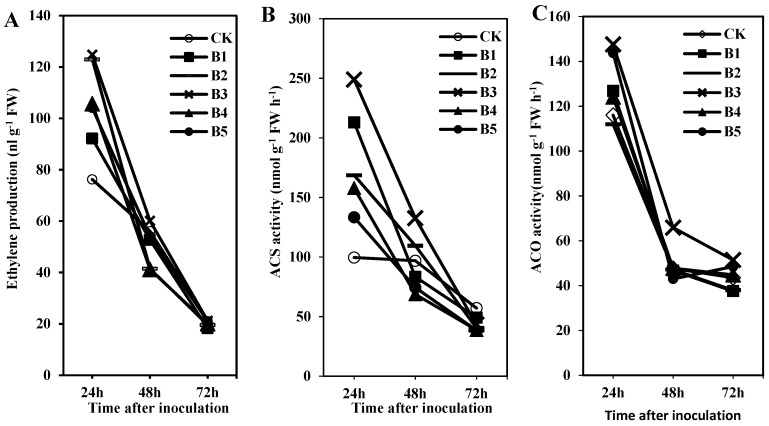
Changes in ethylene production and related enzyme activities of creeping bentgrass during an ISR response induced by BDO. (**A**) ethylene production; (**B**) ACS activity; (**C**) ACO activity. Creeping bentgrass seedlings were treated with different concentrations of BDO (CK = 0 µmol L^−1^, B_1_ = 50 µmol L^−1^, B_2_ = 75 µmol L^−1^, B_3_ = 100 µmol L^−1^, B_4_ = 125µmol L^−1^, B_5_ = 150 µmol L^−1^).

Gaseous ethylene is recognized as an important hormone that regulates and affects plant disease resistance. However, there are few literatures to clarify the detail of creeping bentgrass in response to *R. solani*, especially induction under ISR response to BDO induction. Thus, we measured the change in ethylene accumulation and related enzyme (ACO and ACS) activities in the creeping bentgrass-*R. solani* interaction under the ISR response to the BDO induction. We grew creeping bentgrass seedlings in GC vials to avoid wounding-induced ethylene production during leaf collection. In Figure 1A, ethylene gradually decreased during the 24–72 h after *R. solani* inoculation, and the maximum level occurred in the first 24 h. In addition, in the first 24 h, ethylene production was different among the various BDO treatment concentrations. In the 100 µmol L^−1^ BDO treatment, ethylene accumulation reached a peak at 24 h after the *R. solani* inoculation, which was 124.80 nL g^−1^ FW. However, there was no significant difference in ethylene production 72 h after *R. solani* inoculation with three different BDO concentrations.

ACS and ACO are important enzymes that can synthesize and convert ethylene. As shown in Figure 1B,C, ACS and ACO activity patterns were similar to the ethylene production changes during 24–72 h after *R. solani* inoculation. There were significant decreases in the ACS and ACO activities after the *R. solani* inoculation in creeping bentgrass receiving BDO treatments. After the 100 µmol L^−1^ BDO treatment, the ACS activity reached its peak at 24 h, followed by 48 and 72 h, after the *R. solani* inoculation, with values of 249.02, 132.70 and 43.35 nmol g^−1^ FW h^−1^, respectively. ACO activity was 147.58, 65.87 and 51.45 nmol g^−1^ FW h^−1^, respectively. In general, the changes in ethylene production and related enzyme activities showed significant differences 24 h after *R. solani* inoculation among the five different BDO treatments. However, there was no significant difference 72 h after the inoculation. Additionally, in the 100 µmol L^−1^ BDO treatment, ethylene production and related enzyme activities reached their peak level. Thus, in further analyses, we chose the 100 µmol L^−1^ BDO concentration to analyze the expression levels of ethylene-related genes.

### 2.2. Illumina Sequencing and De Novo Assembly

**Figure 2:**
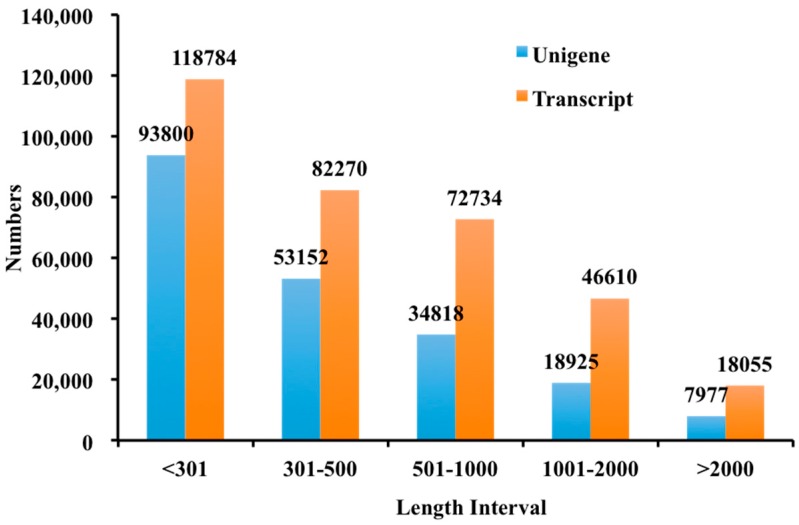
Histogram of the sequence-length distribution of transcripts and unigenes by De novo assembly.

Totally, 954,985,454 paired-end reads were produced for three replicates of CKB24, B24, NB24 and B72, and two technical replicates of each biological replicate, which produced ~150 Gb of data. After removing a small amount of joint pollution, reads containing ploy-N and low-quality reads, the number of cleaned and qualified reads was 921,578,368, and the number of transcripts generated using Trinity (Table 1) was 338,453. Approximately 59.40% of the transcripts were ~200–500 bp (N50 = 1024). In total, 208,672 sequences (61.65% of the total transcripts) were obtained as unigenes with an average length of 557 bp and an N50 of 775 bp. The length distribution of the transcripts and the unigenes are presented in Figure 2.

### 2.3. Functional Annotation of Assembled Unigenes

**Figure 3:**
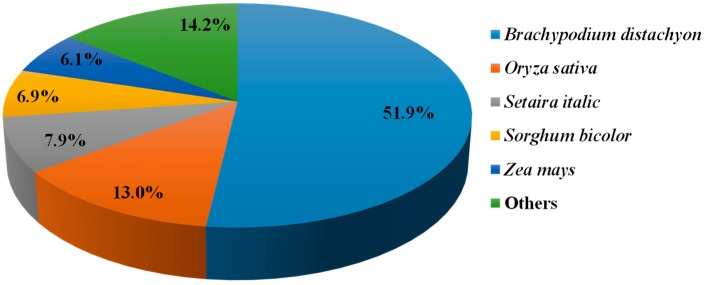
Taxonomic source and distribution (%) of the top results from a BLAST algorithm-based search using creeping bentgrass sequences.

The high-quality unigenes of creeping bentgrass were annotated by alignment with seven public databases. Using BLASTX with a cut-off *E*-value of 10^−5^, in total, 71,824 sequences (34.41%) were similar to proteins in the non-redundant (NR) database. Besides, the assembled unigenes were annotated by alignment with the other six public databases, which include a manually annotated and reviewed protein sequence database (Swiss-Prot), NCBI nucleotide sequences (Nt), GO database, Protein family (Pfam), and euKaryotic Ortholog Groups (KOG). As seen in Table 2, 46,867 unigenes (22.45%) were similar to proteins in the NR database, 39,602 sequences (18.97%) were annotated in the Swiss-Prot database, 50,138 sequences (24.02%) were annotated in the Pfam database, and 57,685 (27.64%) were annotated in the GO database. Totally, 90,004 unigenes (43.13%) were effectively annotated in at least one of the NR, Swiss-Prot, Nt, KOG, Pfam databases and GO, with 6264 unigenes (3%) in seven databases (Table 2). Furthermore, the unigenes of creeping bentgrass were significantly similar to *Brachypodium distachyon* proteins (51.90%), secondly *Oryza sativa* (13.00%) and then *Setaria italic*, *Sorghum bicolor* and *Zea mays* proteins, which accounted for 7.90%, 6.90% and 6.10%, respectively. The other unigenes accounted for 14.20% of the total transcripts (Figure 3).

### 2.4. GO Classification

**Figure 4:**
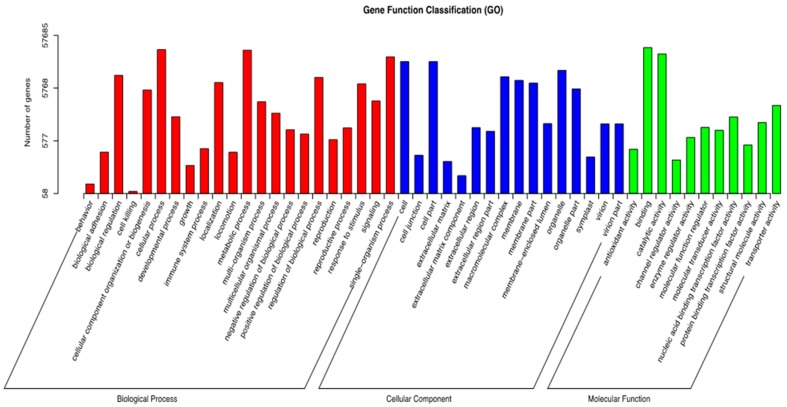
Histogram of gene ontology distributions for the creeping bentgrass transcriptome. Three main functional categories were identified, molecular function, biological process and cellular component.

A GO functional classification for the creeping bentgrass transcriptome was performed. There were 57,685 unigenes annotated into three ontologies (cellular component, biological process, and molecular function) with 49 functional groups (Figure 4). In biological process, ‘single-organism process’ (22,449), ‘cellular process’ (31,019) and ‘biological regulation’ (10,012) were mainly represented. Additionally, in the cellular component, ‘organelle’ (12,555), ‘cell part’ (18,359) and ‘cell’ (18,369) were involved. The ‘catalytic activity’ (25,694) ‘binding’ (33,900), and ‘signaling’ (3267) were highly represented within the molecular function (Figure 4).

**Figure 5:**
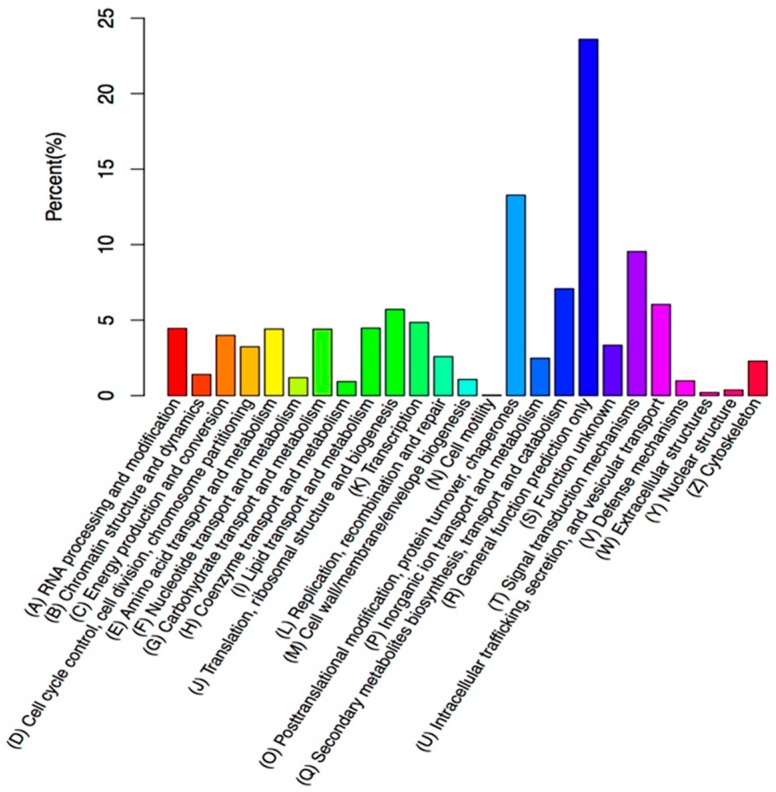
KOG annotation of putative proteins. Totally, 16,393 putative proteins in 25 specific categories.

**Figure 6:**
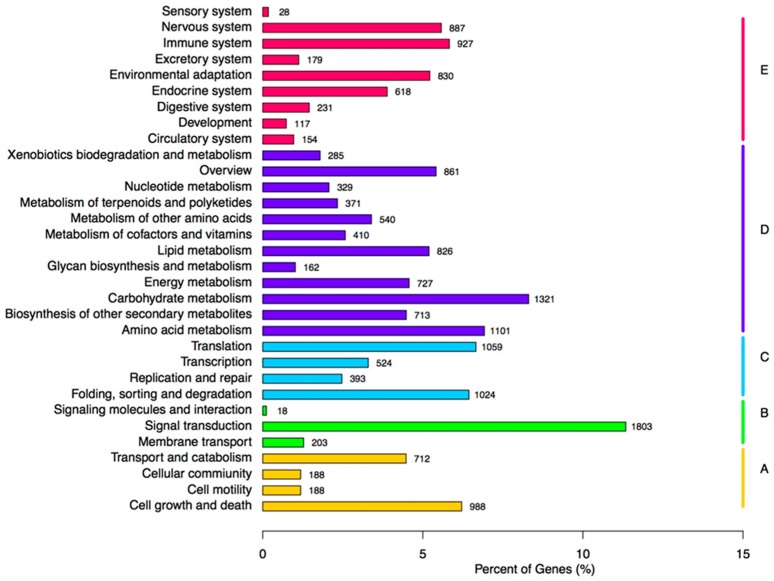
Pathway assignment based on KEGG. (**A**) Cellular Processes; (**B**) Environmental Information Processing; (**C**) Genetic Information Processing; (**D**) Metabolism; (**E**) Organismal Systems.

Furthermore, all unigenes underwent a KOG classification analysis for functional predictions. In total, 16,393 unigenes were assigned to KOG classifications and involved in twenty-five specific categories (Figure 5). Among the twenty-five KOG categories, the three largest group were, ‘general functional prediction only’ (3868; 23.59%), ‘post-translational modification, protein turnover, chaperones’ (2177; 13.28%), and ‘signal transduction mechanisms’ (1565; 9.2%). In addition, ‘cell motility’ had the fewest corresponding genes (Figure 5). The KEGG pathway database was adopted to further identify active biological pathways in creeping bentgrass, which included different active metabolic processes within an organism. The 15,903 annotated unigenes were grouped into 33 canonical reference pathways in the KEGG (Figure 6). Among those, the highest number, 1803 unigenes, was in ‘signal transduction’, followed by 1321 unigenes in ‘carbohydrate metabolism’, 1101 unigenes in ‘amino acid metabolism’, 1059 unigenes in ‘translation’, and 1024 unigenes in ‘folding, sorting and degradation’. It indicated that these were active pathways in creeping bentgrass (Figure 6).

### 2.5. Differential Expression Analysis of Assembled Transcripts

**Figure 7:**
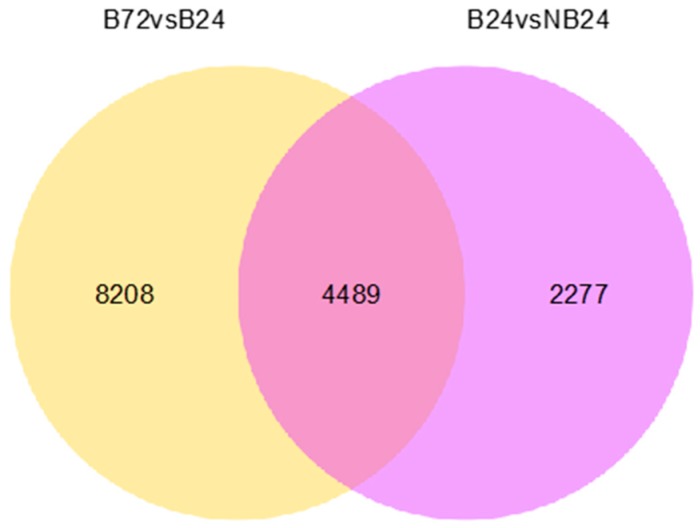
Venn diagram of differentially expressed genes (B24 vs. NB24 and B24 vs. B72). The numbers in each circle denote the total number of different genes in the comparison, and the overlapping sections denote the different shared genes between two combinations.

**Figure 8:**
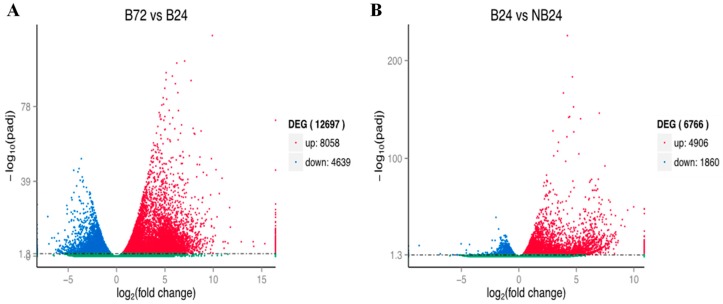
Volcano plots of differentially expressed sequences. ((**A**), the up-regulated or down-regulated genes between B72 and B24; (**B**), the up-regulated or down-regulated genes between NB24 and B24). The abscissa denotes the expressed fold change of genes in different treatments. The ordinate denotes the statistically significant difference degree. Different genes were filtered using padj < 0.05. The scatter points in the diagram denote each gene, and the green dots indicate there was no significant difference. The red and the blue dots are for up-regulated and down-regulated genes, respectively.

The comparison results of Bowtie were assessed by RNA-seq by expectation-maximization [20] to obtain their respective read count numbers, and exchanged them with FPKM. FPKM quantified the transcript level in reads, which provided the comparison of mRNA levels within four treatments. DEGs (*q*-value < 0.005 and |log_2_ (Fold Change)| > 1) were defined as genes with differential expressions as analyzed by read counts in one treatment compared with another treatment. In total, 12,465 unigenes were significantly expressed in four different treatments. As shown in Figure 7, the distribution of expression genes indicated by the Venn diagram among the compared combinations (B24 vs. NB24 and B24 vs. B72). In brief, there were 6766 DEGs between the B24 and NB24 libraries, 4639 DEGs between the B24 and B72 libraries, and 4489 DEGs in all three libraries (Figure 7). Furthermore, as shown in the volcano plots of Figure 8, the up- or down-regulated genes were filtered by padj < 0.05.

### 2.6. Expression Analysis of ET-Responsive and Defense-Related Genes in the BDO-Induced ISR Response

**Figure 9:**
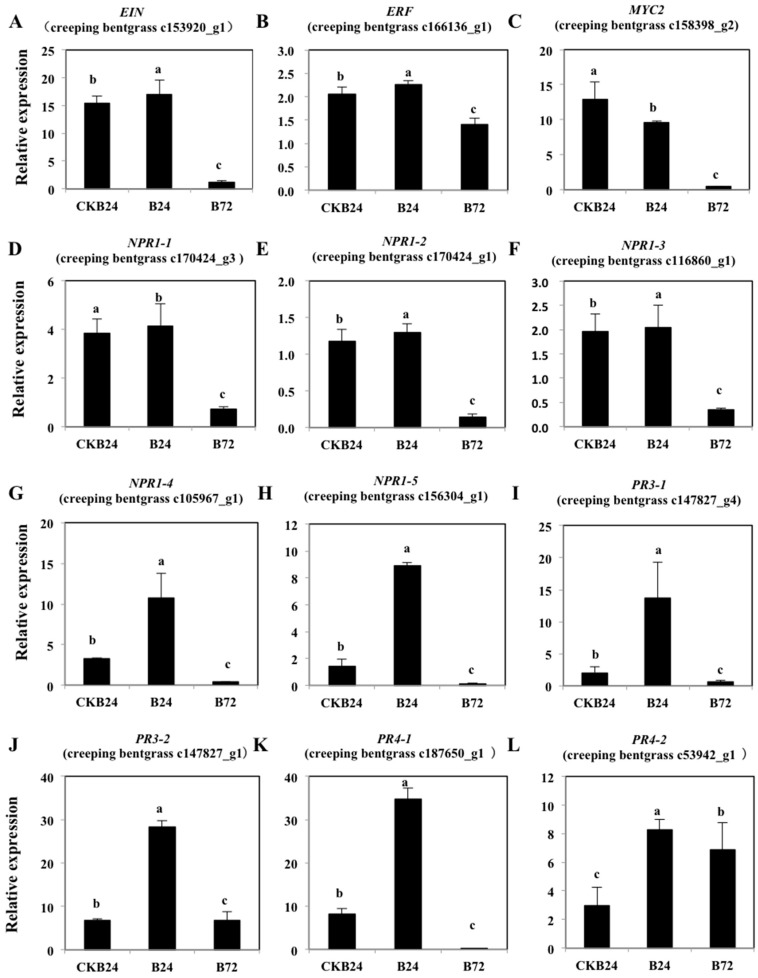
The qRT-PCR analysis of creeping bentgrass ET-responsive genes (*EIN* in subfigure (**A**) , *ERF* in subfigure (**B**), *MYC2* in subfigure (**C**) and *NPR1* in subfigure (**D**–**H**) and defense-related genes (*PR*-*3* in subfigure (**I**,**J**) and *PR*-*4* in subfigure (**K**,**L**)) in the ISR response among different treatment samples (NB24, CKB24, B24 and B72). Results represent mean fold increases (x-fold) in mRNA levels ± standard deviation relative to those of at NB24 treatment) obtained from three independent experiments. Lowercase letters above the columns are used to indicate statistically different groups (*p <* 0.05).

Through RT-PCR assays, relative transcription data were obtained for selected ET-responsive and defense-related genes in the ISR response among different treatment samples (NB24, CKB24, B24 and B72). The 12 target genes were differentially expressed in non-pathogenic and pathogenic seedlings of creeping bentgrass induced by BDO (Figure 9). This assay measured the expression levels of key genes, which were identified in previous transcriptome experiments, involved in the ET signal responsive pathway and defense-related genes. At first, we extracted the data of “response to stress related genes” and “ET signal pathway genes”. The transcriptome sequencing program of creeping bentgrass (*Agrostis stolonifera*) was used in the analysis of eukaryotic nonparametric mRNA sequences. Thus, we got sequence information for several key genes, including *EIN*, *ERF*, *MYC2*, *NPR1-1*, *NPR1-2*, *NPR1-3*, *NPR1-4*, *NPR1-5*, *PR3-1*, *PR3-2*, *PR4-1* and *PR4-2* from “response to stress related genes” and “ET signal pathway genes” (Figure 9).

**Figure 10:**
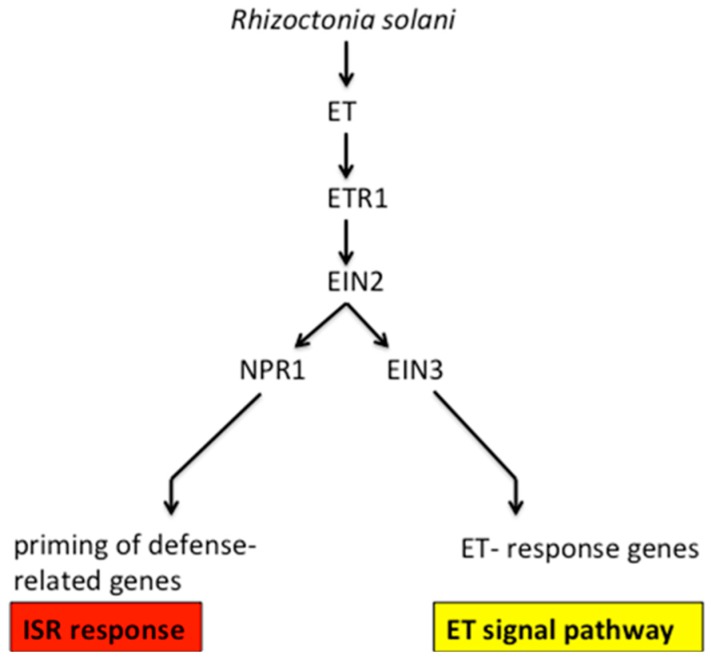
Proposed signal transduction pathways of the ISR and ET.

The expression levels of *EIN* and *ERF* from “ET-responsive genes” was increased at 24 h and decreased at 72 h after *R. solani* infection in BDO-induced ISR reaction compared to non-pathogenic treatments at 24 h (NB24). However, for pathogenic treatments without BDO at 24 h (CKB24), the expression levels of genes raised compared with the NB24 treatment (Figure 9A,B). The important *MYC2* transcription factor, which is a JA/ET antagonistic interaction gene, inhibited the expression of the *EIN* gene. The expression of *MYC2* decreased after pathogenic treatments from 24 h to 72 h, whereas it increased in the CKB24 treatment compared with the NB24 treatment (Figure 9C). We analyzed the expression levels of creeping bentgrass *NPR1* homologs (*NPR1-1*, *NPR1-2*, *NPR1-3*, *NPR1-4* and *NPR1-5*) in NB24, CKB24, B24 and B72 treatments using qRT-PCR (Figure 10D–H). The expression levels of creeping bentgrass *NPR1* homologous genes were highly induced in the B24 treatment while were only slightly induced in B72. Additionally, these genes in the CKB24 treatment have expression levels higher than in the NB24 treatment while was lower than in the B24 treatment. In addition, we also analyzed the expression levels of pathogenesis-related (*PR*) genes (*PR3-1*, *PR3-2*, *PR4-1* and *PR4-2*) of creeping bentgrass in NB24, CKB24, B24 and B72 treatments (Figure 10I–L). The expression levels of these genes showed the same trends as the *NPR1* genes, which were highly induced in the B24 treatment and slightly induced in B72. Compared with the expression levels of these genes in the NB24 treatment, they were highly induced in the CKB24 and B24 treatments. Thus, the positive responses of creeping bentgrass to *R. solani* may occur gradually at 24 h. The expression levels of the above genes were slightly induced after 72 h. At 3th day, we have observed the brown blotch symptoms (see “Materials and Methods”). The disease-resistance genes induction and physiological characteristics of creeping bentgrass was earlier than pathogenic symptoms appearance, so we assay genes expression in 24–72 h (1–3 day) time points, which was consistent with above conclusion.

## 3. Discussion

In this study, 208,672 unigenes were found for creeping bentgrass. In total, 51.90% of the transcripts had hits from a BLASTx algorithm-based search against *B. distachyon* proteins by comparison with the NR database. It suggested that creeping bentgrass have close relation to Brachypodium, which is a C3 grass and usually taken as a model plant in grass studies [21]. Additionally, creeping bentgrass had a 13% similarity with *O. sativa* (Figure 3). Thus, we hypothesized that *B. distachyon* and *O. sativa* are closely related species to creeping bentgrass. In further research, the gene recourses of above two plants can be used the reliable references. Additionally, we examined diseases-resistance and ISR-response genes expression differences among NB24, CKB24, B24 and B72. These factors are critical in selecting DEGs based on the GO annotations and enriched KEGG terms for signal transduction to further understand the role of the ET signal pathway in ISR disease-resistance response. In this research, 15,903 annotated unigenes were grouped into 33 reference canonical pathways in the KEGG (Figure 6), among which, the greatest amount, 1803 unigenes, were found in ‘signal transduction’.

Besides their functions in plant growth and development, plant hormones are important signal molecules and play complex roles in the signal pathway response in plant–pathogen recognition [22]. In our research, 31 genes were plant hormone signal transduction molecules, including in the plant-pathogen signal transduction network in the B24 vs. NB24 treatment comparison. Among these plant hormones, ethylene plays important roles in developmental and physiological processes in plants including ripening, leaf and flower senescence, seedling emergence, organ abscission, and responses to adverse abiotic and biotic stresses [23]. Under a diversity of stress induction, such as drought, pathogen infection, wounding and oxidative stresses, ethylene production is induced and increased in some plant species [24,25]. Through determination and analysis, 13 genes were the ethylene-responsive genes. In the previous research, early ethylene production enhances responses to plant-pathogen interactions and positively induces the defense reactions [26]. For example, ethylene was found to be related disease-resistance in carrot [27]. And ethylene involvement disease resistance is mostly based on the induction of a set of defense-related genes after pathogen attack in *Arabidopsis thaliana*.

At present detailed measurements of ethylene biosynthesis and related enzymes in turfgrass resistance are lacking, especially in the ISR reaction pathway induced by BDO. In our study, we characterized changes in the important ethylene biosynthetic enzymes and ethylene concentrations after BDO treatments (Figure 1). Our conclusion that the ethylene production and related enzymes (ACS and ACO) activities showed similar trends in ISR response induced by BDO, which gradually decreased from 24 to 72 h after *R. solani* inoculation, and were greatest in the first 24 h in creeping bentgrass after BDO treatment. Based on analyses of ethylene biosynthesis, we concluded that ethylene positively influences creeping bentgrass resistance against *R. solani* in a short time after inoculation, and ethylene molecule triggers ethylene signal pathway to participate in ISR response induced by BDO, which is a rapid process. It is interesting to analyze ethylene biosynthetic characters within 24 h inoculation on creeping bentgrass in future study.

We explored the relevance of ethylene signal pathway-responsive genes and BDO-induced ISR resistance genes. The results of RT-PCR suggested that the expression of ET-responsive and defense-related genes in the BDO-induced ISR response among different treatments (Figure 9). The *EIN* and *ERF* from “ET-responsive genes” are very important genes in the ethylene signal conduction pathway, and are upstream of five ethylene receptors, *ETR1*, *EIN4*, *ERS2*, *ERS1* and *ETR2*. First, ethylene binds to the membrane-bound receptor, then, receptor signaling is inactivated, allowing the downstream signaling of the central regulatory protein EIN2, and resulting in the activation of diverse transcriptional regulatory factors and defense-related genes. EIN2 has important effects in the ethylene signal pathway, and the loss of the *EIN2* gene function leaves the plant completely insensitive to ethylene [28]. Through a transcriptional cascade reaction, EIN2 regulates EIN3 and EIL1, and then, EIN3 binds to the EIN3-binding site in the promoter of ERF1, and then activates ethylene responses [17]. ERF, the ethylene response factor, encodes a transcriptional activator to promote several downstream ethylene-responsive genes (Figure 10). Additionally, MYC2 and ERF1, the transcription factors, participate in the ET and JA signaling pathways and activated defense-related genes, which are responsive to both ET and JA. In our study, the expression levels of *EIN* and *ERF* increased and reached their maximum levels in the first 24 h after *R. solani* infection in the BDO-induced ISR reaction compared with non-pathogenic treatments at 24 h (NB24) (Figure 9A,B). This conclusion is consistent with the changes in important ethylene biosynthetic enzymes and in the ethylene concentration during the process. And it also demonstrates the short-time effect of ethylene molecule on disease-resistance of creeping bentgrass. The ethylene reaction is needed for the development and expression of ISR [29]. For example, in ET insensitive mutants of *Arabidopsis*, the ET receptor gene *etr-1* is susceptible to the non-pathogenic oomycete *Pythium sylvaticum*. A similar phenomenon, in which the ET-responsive mutant *etr1-1*, *ein2-1-ein7* could not express ISR when root is infected by strain WCS417, has been observed [30]. However, several studies have demonstrated that the defense-regulating hormone jasmonic acid or salicylic acid is also required for the expression of defensive activities in different plant species [31]. For example, in tomato or tobacco, the salicylic acid (SA) dependent defense pathway is relevant to resistance against *Botrytis cinerea* and the powdery mildew fungus *Oidium neolycopersici* [32]. Thus, the plant’s induced resistance signaling is a complex crosstalk network, and the ET-dependent ISR response is also important in the induction of plant disease-resistance.

In the SA-mediated SAR reaction of *Arabidopsis*, NPR1 is a crucial regulatory protein and impacts the SAR pathway downstream of the SA signal pathway. During SAR, NPRI induces the *PR* gene expression, and even after inducing by SA, BTH or INA, *NPR1* expression levels increase [33,34]. Pieterse et al. [29] determined that *NPR1* also participates in the jasmonate- and ethylene-dependent ISR. The *npr1* mutant was unable of generating a WCS417r-mediated ISR response. A further study of ISR responses unveiled that NPR1 is the downstream of the ET- and JA-dependent signal-transduction pathway steps (Figure 10). Our study found that the expression levels of creeping bentgrass *NPR1* homologous genes were highly induced in the B24 treatment, but only slightly induced in B72. Additionally, the expression of *EIN* and *ERF* genes from “ET-responsive genes” and the changes in important ethylene biosynthetic enzymes and ethylene concentrations in the process were similar. These indicate a key role for the NPR1 protein in regulating ET-dependent defense pathways, and BDO-induced ISR was an NPR1-dependent defense response (Figure 9).

In *Arabidopsis*, ET applied exogenously activates certain series of defense-related genes and induces resistance. To further research the molecular mechanism of rhizobacteria-mediated ISR induced by BDO, we focused on the PR proteins because their accumulation may be related to induced disease resistance. The expression levels of creeping bentgrass *PR3* and *PR4* genes were highly induced in the B24 treatment but only slightly induced in B72. This conclusion is consistent with the changes in the important ethylene biosynthetic enzymes, ethylene concentrations and “ET-responsive genes” in the process. In ISR response of *Arabidopsis*, the JA/ET response gene *PR4* is elicited by the JA/ET signal and expressed, and the expression product induces resistance to multiple rhizobacteria [19,35], and some studies have reported ET and JA co-regulate series of genes encoding PR proteins, including *PR-3*, *PR-4* and *PR-1* [36]. Cortes-Barco et al. [5] pointed that BDO inspires the expression of the disease-resistance genes *AsAOS1*, *AsO-PR4* and *AsGns5*. However, some previous research showed that radish roots treated with ISR-inducing WCS417r did not accumulate PR protein, even though disease resistance was enhanced [37]. Analogously, the expression of SAR marker genes *PR-1*, *PR-2* and *PR-5* and WCS417r-mediated ISR was not active, but plants showed enhanced resistance in *Arabidopsis* [38]. In short, we preliminarily discussed the intermediate substances in the signaling pathway, as well as the relationships between the expressed BDO-induced ISR disease-resistance genes and the response genes of the ethylene signal pathway on creeping bentgrass. It must have more complex mechanism between ISR response and ethylene signal pathway on turfgrass, which needs further investigation. Our findings present a genetic basis for systemic resistance of creeping bentgrass through transcriptomic analysis and our study provides a theoretical and practical basis for the improvement of turfgrass disease resistance and quality.

## 4. Materials and Methods

### 4.1. Plant Growth Conditions and Treatments

**Figure 11:**
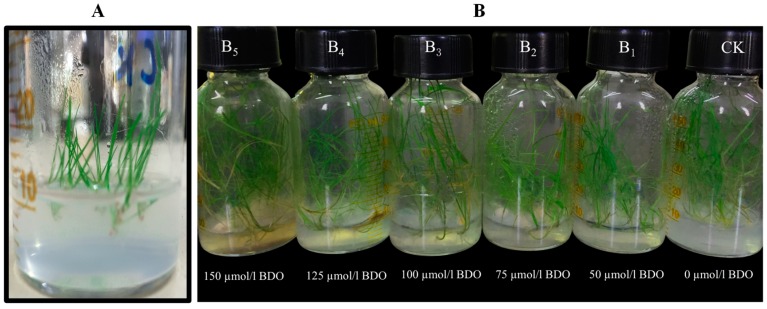
(**A**) About 20 seedlings per vial were grown in 50-mL gas chromatography vials with 10 mL MS medium; (**B**) Creeping bentgrass seedlings were treated with different concentrations of BDO (CK = 0 µmol L^−1^, B_1_ = 50 µmol L^−1^, B_2_ = 75 µmol L^−1^, B_3_ = 100 µmol L^−1^, B_4_ = 125µmol L^−1^, B_5_ = 150 µmol L^−1^) 72 h after inoculation.

Seeds from creeping bentgrass ‘PennA-4′ were grown by modified method of Kroes et al. [39]. The surface of seeds was sterilized with 70% ethanol for 1 min, followed by 15% sodium hypochlorite for 5 min. After five rinses with sterile water for 10 min, seeds were dried with filter paper. Seeds were then sown in 50-mL gas chromatography (GC) vials with 10 mL of MS medium containing different concentrations of BDO (50 µmol L^−1^, 75 µmol L^−1^, 100 µmol L^−1^, 125 µmol L^−1^ and 150 µmol L^−1^). Approximately 20 seedlings per vial (Figure 11) were placed at 22 °C under continuous light (100 µE m^−2^ s^−1^) in a growth chamber. The control was not received a BDO treatment (CK). The experimental materials were seedlings which is twelve-day-old grown in GC vials.

*Rhizoctonia solani* (#3.2888 from China General Microbiological Culture Collection Center) isolates were grown in liquid potato dextrose medium (200 g L^−1^) in triangular flask with shaking for 4 day at 25 °C. Mycelia were removed and washed with sterile water three times, added to sterile water after grinding in a mortar. Concentration of the bacterial sample was a final OD_340_ of 0.8. Roots of seedlings were directly sprayed with 2 mL of the *R. solani* bacterial fluid under sterile conditions.

In our experiment, brown blotch symptoms were observed within 3–5 day post-inoculation on creeping bentgrass seedlings. The mycelia grew but had severe symptoms. After 24, 48 and 72 h post-inoculation, ethylene and related enzymes were measured. The 100 µmol L^−1^ BDO-treated materials were removed from the medium, and the leaves were cut with a sharp blade, cleaned with sterile water and dried. Leaves were placed in 5-mL tubes, frozen in liquid nitrogen and stored at −80 °C for further analysis. In this experiment, four seedling treatments, CKB24 (inoculated-rhizobacteria in MS medium without BDO for 24 h), B24 (inoculated-rhizobacteria in MS medium with 100 µmol L^−1^ BDO for 24 h), NB24 (no rhizobacteria in MS medium with 100 µmol L^−1^ BDO for 24 h) and B72 (inoculated-rhizobacteria in MS medium with 100 µmol L^−1^ BDO for 72 h), were performed for transcript profiling. 

### 4.2. Ethylene Measurement

The creeping bentgrass seedlings in GC vials were inoculated with rhizobacteria and immediately capped. At the measurement times, the ethylene levels in the headspace were measured using gas chromatography (Varian 450-GC, Palo Alto, CA, USA) by the modified method of Kim et al. [40]. 

### 4.3. 1-Aminocyclopropane-1-Carboxylic Acid Synthase (ACS) and 1-Aminocyclopropane-1-Carboxylic Acid Oxidase (ACO) Activity Assays

Leaf tissue (2 g) was homogenized at 0 °C with 100 mM Hepes extraction buffer (5 mL; 4 mM dithiothreitol, 5 mM ethylene diamine tetraacetic acid, 0.5 μM pyridoxal phosphate, 1% polyvinyl pyrrolidone and 10% glycerol, pH 8.5). The homogenates were centrifuged at 25,000× *g* at 4 °C for 20 min. The obtained supernatant was desalted with 2 mM Hepes (0.1 mM dithiothreitol and 0.2 μM pyridoxal phosphate, pH 8.5) for 12 h. The dialysate was used in the ACS activity assay. ACS activity was measured by incubating 1 mL of the enzyme preparation with 0.5 mL of 60 μM S-adenosylmethionine at 30 °C for 2 h, and 0.2 mL 10 mM HgCl was added to end the reaction [41]. Then, 1 mL of reaction mixture was moved to a 10-mL blood collection tube, then added to 200 μL 5% NaClO–saturated NaOH (2:1, *v*/*v*). It was whirled and shaken for 5 s, which was repeated after 3 min. Released gas (1 mL) was assayed for ethylene production. The amount of ethylene (nmol) produced per g of fresh weight (FW) per h was used to express the ACS enzyme’s activity level. 

Using a previous method [42] with some changes, leaf tissue (2 g) was homogenized at 0 °C with 0.1 M Tricine buffer (10% glycerol and 30 mM sodium ascorbate, pH 7.5). Homogenates were centrifuged at 20,000× *g* at 4 °C for 20 min. Then, 3 mL obtained supernatant was moved to a 10-mL blood collection tube, 0.35 mL 0.2 M Tricine buffer (20% glycerol, 0.2 mM FeSO_4_, 60 mM sodium ascorbate, 40 mM NaHCO_3_, pH 7.5) was added, then 0.35 mL 1 mM ACC was added for 30 min at 30 °C, and the production of 1 mL ethylene was determined. The amount of ethylene (nmol) produced per g of FW per h was used to express the ACO activity level.

### 4.4. Total RNA, mRNA Isolation and Library Preparation for Transcriptome Sequencing

Creeping bentgrass leaf blades were gathered from CKB24, B24, NB24 and B72 with three repetitions to minimize transcriptional variation caused by the changes of the plant’s growth stage or physiology. Leaves were gathered for RNA extraction and frozen in liquid nitrogen. By using TRIzol reagent (Qiagen, Hilden, Germany) total RNA was extracted, and purified by using an RNeasy Plant Mini kit (Qiagen) following the manufacturer’s instructions. By using a NanoPhotometer spectrophotometer (IMPLEN, Munich, CA, USA), RNA purity was tested. An Agilent 2100 system (Agilent Technologies, Palo Alto, CA, USA) was used to test the quality and integrity of the RNA.

### 4.5. Library Preparation for Transcriptome Sequencing

The 2-μg RNA samples were prepared as input materials. Sequencing libraries were produced using NEBNext Ultra^TM^ RNA Library Prep Kit for Illumina (NEB, San Diego, CA, USA), and each sample attribute sequences for index codes. In brief, the poly-T oligo-attached magnetic beads were used to purify mRNA. First-strand cDNA synthesis was performed using random primers and reverse transcriptase. Second-strand cDNA was then synthesized using RNase H and DNA Polymerase I. The AMPure XP system (Beckman Coulter, Beverly, MA, USA) was used to purify the library fragments to isolate cDNA fragments in length of ~200–300 bp. Then, adaptor-ligated, size-selected cDNA was mixed with 3 μL USER Enzyme (NEB) for 15 min at 37 °C, then 5 min at 95 °C before PCR. Phusion High-Fidelity DNA polymerase, Index (X) Primer and Universal PCR primers were used to purify and enrich the products by PCR to create the final cDNA library. Using HiSeq 4000 PE Cluster Kit Box1 (Illumina, San Diego, CA, USA), the clustering of the index-coded samples was produced on a cBot Cluster Generation System. Then, on an Illumina HiSeq 4000 platform the multiplexed library preparations were sequenced and 150-bp paired-end reads were produced. RNA-seq read data were placed on the National Center for Biotechnology Information (NCBI) Sequence Read Archive under accession number SRR5658390.

### 4.6. Preprocessing and De Novo Assembly

Using perl scripts, raw reads in fastq format were processed. To remove a small amount of joint pollution, low-quality reads (Q < 20) and reads containing poly-N (N > 10%) were eliminated, and the qualified reads were assembled. In the meantime, the GC content and Q20, Q30 of the clean data were calculated. The high-quality clean data is used to perform all of the downstream analyses. Because there is no reference creeping bentgrass genome, using the Trinity software package (v2012-10-05 http://trinityrnaseq.github.io) clean reads were joined de novo with min_kmer_cov set to 2 by default and all other parameters set to default according to a previous description by Grabherr et al. [43].

### 4.7. Quantification of Gene Expression Levels and Differential Expression Analysis

Gene expression levels were assessed using the RNA-seq by expectation-maximization alignment of each sample [20]. Clean data were mapped back onto the collected transcriptome libraries. From the mapping results, a read count for each gene was obtained. Differential expression analyses of four treatments (two biological replicates per treatment) were done using the DESeq R program package (1.10.1). Using the Benjamini–Hochberg’s method to multiple comparisons [44] the *p*-values were assessed. By DESeq Genes with an adjusted *p*-value < 0.05 were found, and assigned as differentially expressed. Using fragments per kilobase of transcript per million fragments mapped (FPKM), the levels of expression were quantified.

### 4.8. Unigene Annotation and Classification

Determining the gene functions of the transcripts were conducted based on NR, KOG, SwissProt, KEGG databases using the BLASTALL package (release 2.2.28) [45] from the NCBI with a significant threshold of *E*-value ≤ 10^−5^. The statistical enrichment of differential expression genes (DEGs) was checked using KOBAS software in the KEGG pathways [46]. Gene Ontology (GO) consists of three parts, cellular component, biological process, and molecular function, for the functional categorization using Blast2go v2.5 software (Biobam, Spain). The *E*-value filter for the GO annotation was 1 × 10^−6^. Using gene IDs the corresponding GO terms in functional categories can be identified. GO classification associations, combined with statistically transcripts, are presented.

### 4.9. Quantitative Real-Time PCR Analysis

The differential expression levels of 12 unigenes under BDO induction of the ISR disease-resistance reaction were analyzed by real-time qRT-PCR. These genes were protective proteins, ethylene response factors and transcription factors in the ET signal pathway. Extraction of total RNA was performed using the TRIzol reagent (Qiagen). First-strand cDNA was synthesized from 500 ng of total RNA using a FastQuant RT Kit (Qiagen). The 1 µL of cDNA was used for qRT-PCR. The RT-PCR analysis was performed using SuperReal PreMix Plus (SYBR Green, Qiagen). The Actin gene was chosen as an internal standard, and the relative expression of each target gene was obtained using the 2^−ΔΔCT^ method. For each of four treatments, CKB24, B24, NB24 and B72, three independent biological replicates were performed. Each biological replicate included two technical replicates for the qRT-PCR analysis. The names of the unigenes and primer sequences are listed in Supplementary Table S1.

### 4.10. Statistical Analysis

At least, three independent biological replicates were performed independently for transcriptomic experiments, which were at multiple time points. Three independent repetitions were performed for single time point experiments. For qRT-PCR, three independent biological replicates were performed and each biological replicate include two technical replicates. The statistical analysis was performed by using the Student’s *t*-test. A one-way analysis of variance (ANOVA) was performed on the experimental time point data set (*p* < 0.05).

## Figures and Tables

**Table 1:** Assembled transcripts and unigenes of all creeping bentgrass samples.

Total raw reads	954,985,454
Total clean reads	921,578,368
GC content (%)	54.59
Q20 bases (%)	97.41
Total transcripts	338,453
Total length of transcripts (bp)	227,642,178
Transcript mean length (bp)	673
Transcripts with N50 length (bp)	1024
Total unigenes	208,672
Total length of unigenes (bp)	116,222,950
Unigenes mean length (bp)	557
Unigenes with N50 length (bp)	775

**Table 2:** Annotation summary of creeping bentgrass unigenes.

	Number of Unigenes	Percentage (%)
Annotated in NR	71,824	34.41
Annotated in NT	46,867	22.45
Annotated in KO	15,903	7.62
Annotated in SwissProt	39,602	18.97
Annotated in PFAM	50,138	24.02
Annotated in GO	57,685	27.64
Annotated in KOG	16,393	7.85
Annotated in all Databases	6264	3
Annotated in at least one Database	90,004	43.13
Total Unigenes	208,672	100

## Supplementary Materials

Supplementary materials are available online. Supplementary Table S1: The primer pairs for RT-PCR. (XLSX). Supplementary Table S2: Changes in ethylene production and related enzyme (ACO and ACS) activities of creeping bentgrass during an ISR response induced by BDO.
