# Supplementary material for: Transcript Profiling and Gene Identification Involved in the Ethylene Signal Transduction Pathways of Creeping Bentgrass (Agrostis stolonifera) during ISR Response Induced by Butanediol
Source: Molecules. 2018 Mar 20;23(3):706. doi: 10.3390/molecules23030706 (PMC6017539; doi:10.3390/molecules23030706)
Supplement: Supplementary file 1 [file molecules-23-00706-s001.pdf]

Supplementary Table 1 The primer pairs for RT-PCR

**Transcript number**	**Sequences (from 5' to 3')**		**Expected size (bp)**
c153920_g1	Forward	CCACGAGACACTGGACATGG	171
	Reverse	CCGCCGAAGAAGTACTCCAC	
c166136_g1	Forward	GCAAGGTTGTTGACTGCTGG	186
	Reverse	CAACAGTGCCGAAGAAGCTG	
c158398_g2	Forward	ATGAACCTGTGGACGGACGA	159
	Reverse	CTGGAGCGTGTCATGGTTGA	
c170424_g3	Forward	TCTGCAAGTGCGATGTCCAG	194
	Reverse	CCCGAAGACATGGTCTCTCCTA	
c116860_g1	Forward	GAGCTCGGCATTCGGATCTTA	150
	Reverse	CAGCCTGTTCCAGCATCTCA	
c105967_g1	Forward	TTGGTCGACTCAGGATGCAC	173
	Reverse	GACTACCCAGGTGTTGACGG	
c156304_g1	Forward	GAAGCTGCTCGAAAGATGCC	150
	Reverse	GGAAAGCCGTTATCTTCAGGTG	
c170424_g2	Forward	TTACCTGTACAGTGGCCGTG	172
	Reverse	ATCACGCTGGAAGAGGTTGG	
c147827_g4	Forward	TGTTCATCCAGAACCAGAGCG	198
	Reverse	ACTGCGACAACAAGAACACG	
c147827_g1	Forward	CCTAGTTATGCACACGCGGA	156
	Reverse	CGGGTGAGCTACTACAGGGA	
c187650_g1	Forward	AGAGGTATCGGCAATGGAGG	158
	Reverse	TCTCCGGGTTGTACAGGTTG	
c53942_g1	Forward	TCCCAGTTGATATTCTGTGGGC	177
	Reverse	CTTCCTGCCACTGACCATAGC	
actin	Forward	CAGATCATGTTCGAGACCTTC	200
	Reverse	GACGGTGTGGCTGACACCAT	

Supplementary Table S2 Changes in ethylene production and related enzyme (ACO and ACS) activities of creeping bentgrass during an ISR response induced by BDO.

BDO concentration ($\mu mol\ L^{-1}$)	Ethylene production after inoculation ($nl\ g^{-1}$ FW)			ACO activities after inoculation ($nmol\ g^{-1}$ FW h^{-1})			ACS activities after inoculation ($nmol\ g^{-1}$ FW h^{-1})		
	24h	48 h	72 h	24h	48 h	72 h	24h	48 h	72 h
0	76.25±4.47^{d}	56.10 ± 6.23^{c}	20.95 ± 1.62^{a}	116.03 ± 3.42^{c}	47.67 ± 0.89^{b}	43.53 ± 0.47^{c}	99.62 ± 1.45^{f}	96.98 ± 1.45^{c}	57.17 ± 2.44^{a}
50	92.17 ± 6.56^{c}	52.71 ± 1.50^{b}	18.30 ± 0.86^{b}	126.80 ± 3.97^{b}	46.73 ± 0.33^{b}	37.55 ± 1.84^{d}	213.08 ± 9.17^{b}	83.46 ± 1.15^{d}	49.19 ± 0.75^{b}
75	122.96 ± 2.80^{a}	41.52 ± 1.50^{c}	19.63 ± 0.68^{ab}	112.00 ± 4.44^{c}	47.15 ± 1.00^{b}	37.99 ± 0.97^{d}	168.73 ± 7.97^{c}	109.66 ± 4.37^{b}	40.58 ± 0.90^{c}
100	124.80 ± 0.91^{a}	60.15 ± 1.13^{a}	21.21 ± 0.47^{a}	147.58 ± 2.67^{a}	65.87 ± 0.57^{a}	51.45 ± 0.16^{a}	249.02 ± 6.47^{a}	132.70 ± 4.31^{a}	43.35 ± 0.62^{c}
125	105.72 ± 7.48^{b}	41.22 ± 2.73^{c}	20.12 ± 0.46^{a}	124.14 ± 2.21^{b}	47.82 ± 0.75^{b}	44.83 ± 0.34^{c}	157.90 ± 2.71^{d}	68.76 ± 4.36^{f}	38.76 ± 1.86^{c}
150	104.10 ± 1.57^{b}	54.08 ± 1.54^{b}	21.06 ± 0.39^{a}	143.74 ± 3.71^{a}	43.02 ± 1.30^{c}	48.49 ± 1.89^{b}	133.41 ± 4.78^{e}	74.47 ± 0.84^{e}	38.55 ± 6.89^{c}

Note: Values represent mean ± standard error of three independent experiments. Different lowercase letters indicate significant at $P< 0.05$ level at the same time point with different BDO treatment concentrations.
